# Treatment of long-segment Barrett’s adenocarcinoma by complete circular endoscopic submucosal dissection: a case report

**DOI:** 10.1186/s12876-018-0743-9

**Published:** 2018-01-19

**Authors:** Miki Kaneko, Akira Mitoro, Motoyuki Yoshida, Masayoshi Sawai, Yasushi Okura, Masanori Furukawa, Tadashi Namisaki, Kei Moriya, Takemi Akahane, Hideto Kawaratani, Mitsuteru Kitade, Kousuke Kaji, Hiroaki Takaya, Yasuhiko Sawada, Kenichiro Seki, Shinya Sato, Tomomi Fujii, Junichi Yamao, Chiho Obayashi, Hitoshi Yoshiji

**Affiliations:** 1 Third Department of Internal Medicine, Nara Medical University, 840 Shijo-cho, Kashihara, Nara, 634-8522 Japan; 2 Department of Endoscopy, Nara Medical University, 840 Shijo-cho, Kashihara, Nara, 634-8522 Japan; 3 Department of Diagnostic Pathology, Nara Medical University, 840 Shijo-cho, Kashihara, Nara, 634-8522 Japan

**Keywords:** Barrett esophagus, Adenocarcinoma of esophagus, Endoscopic submucosal dissections, Esophageal stricture

## Abstract

**Background:**

We present the first description of *en bloc* endoscopic submucosal dissection (ESD) for total circumferential Barrett’s adenocarcinoma, predominantly of the long-segment Barrett’s esophagus (LSBE), with a 2-year follow-up and management strategies for esophageal stricture prevention.

**Case presentation:**

A 59-year-old man was diagnosed with LSBE and Barrett’s adenocarcinoma by esophagogastroduodenoscopy (EGD). A 55-mm-long circumferential tumor was completely resected by ESD. Histopathology revealed a well-differentiated adenocarcinoma within the LSBE superficial muscularis mucosa. For post-ESD stricture prevention, the patient underwent an endoscopic triamcinolone injection administration, oral prednisolone administration, and preemptive endoscopic balloon dilatation. Two years later, there is no evidence of esophageal stricture or recurrence.

**Conclusions:**

ESD appears to be a safe, effective option for total circumferential Barrett’s adenocarcinoma in LSBE.

## Background

Recently, there has been an increased incidence of Barrett’s adenocarcinoma and its resection by endoscopic submucosal dissection (ESD) has been reported [1]. Barrett’s esophagus originates from gastroesophageal reflux disease (GERD) and occasionally progresses to esophageal adenocarcinoma. A local resection of cancerous lesions in the Barrett’s esophagus mucosal layer (T1a) is recommended as a therapeutic option because it is minimally invasive. ESD is common in Japan because it allows *en bloc* resection of large lesion [2]; however, GERD-induced submucosal fibrosis beneath the cancerous lesion often makes ESD difficult to perform. A lesion occupying the Barrett’s esophagus circumference poses therapeutic difficulties and has a high risk of post-treatment esophageal stenosis. No reliable methods currently exist for esophageal stricture prevention after complete circular ESD for Barrett’s adenocarcinoma. We describe a case of long-segment Barrett’s esophagus (LSBE), which was mostly replaced by an early esophageal adenocarcinoma, treated by complete circular ESD and followed up for 2 years without esophageal stricture or recurrence.

## Case presentation

**Fig. 1:**
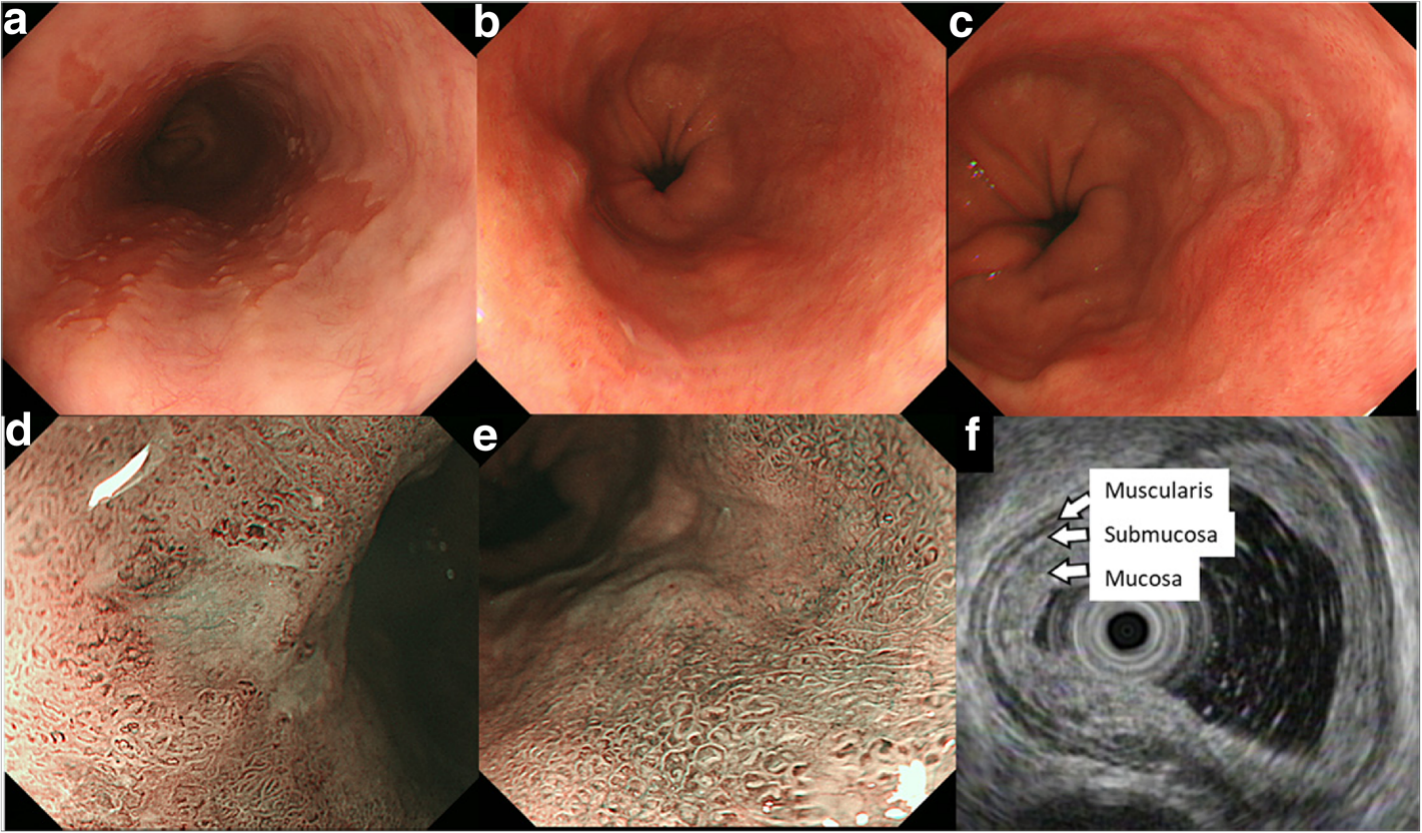
Endoscopic findings. **a** Long-segment Barrett’s esophagus (C4/M5). **b** A reddish and depressed lesion noted at the 7 o’clock position. **c** Rough surface and uneven redness observed. **d**, **e** Irregular structural and vascular patterns detected by magnifying endoscopy with narrow band imaging. **f** Endoscopic ultrasonography (20 MHz) showing the tumor in the mucosal layer with a normal submucosal layer (arrows)

**Fig. 2:**
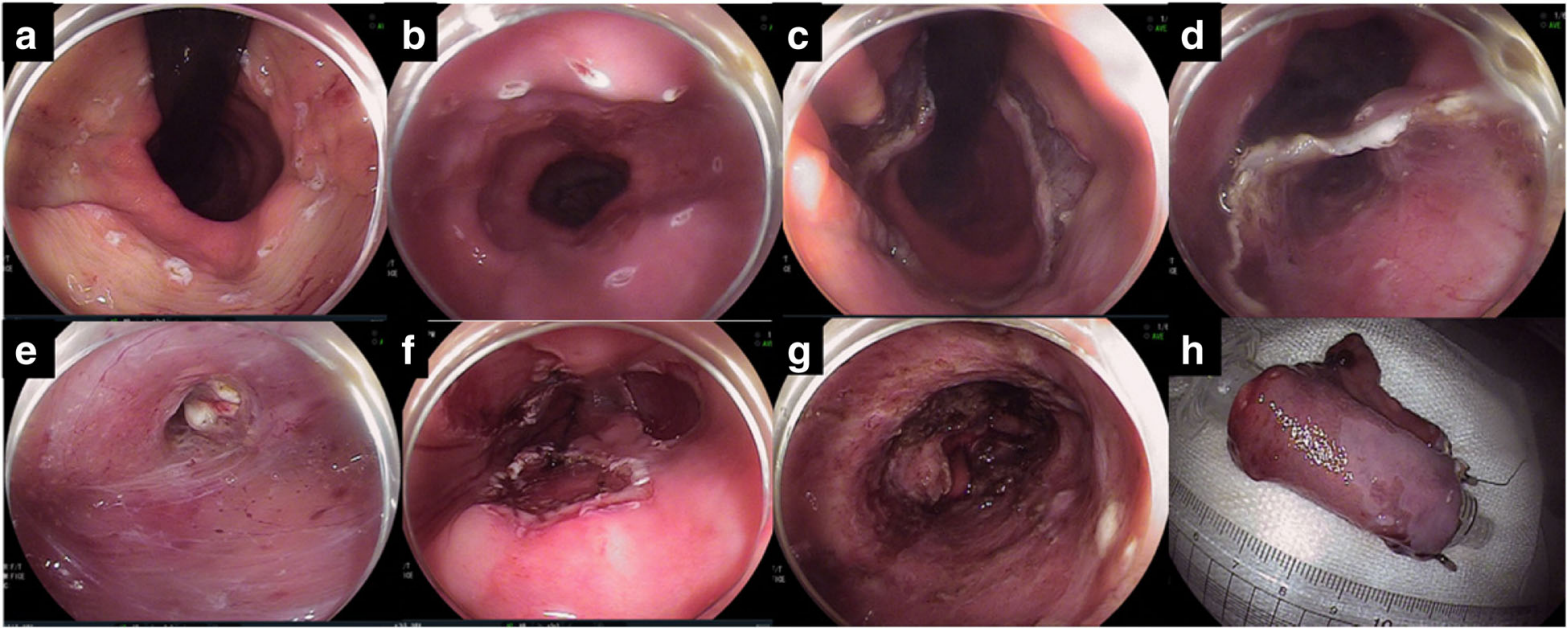
Endoscopic submucosal dissection (ESD). **a** Anal extent of resection. **b** Adoral extent of resection: 1-cm margin from the SC junction, taking into consideration the progression under the squamous epithelium. **c** Circumferential incision at the caudal end of the lesion. **d** Creation of the entrance of a submucosal tunnel at the cranial end of the lesion. **e** Tunnel creation by submucosal dissection. The upper side of the image is the mucosa and the lower side is the muscle. **f** Creation of three submucosal tunnels. **g** Complete circular ESD was performed. **h** *En bloc* resected cylindrical specimen

**Fig. 3:**
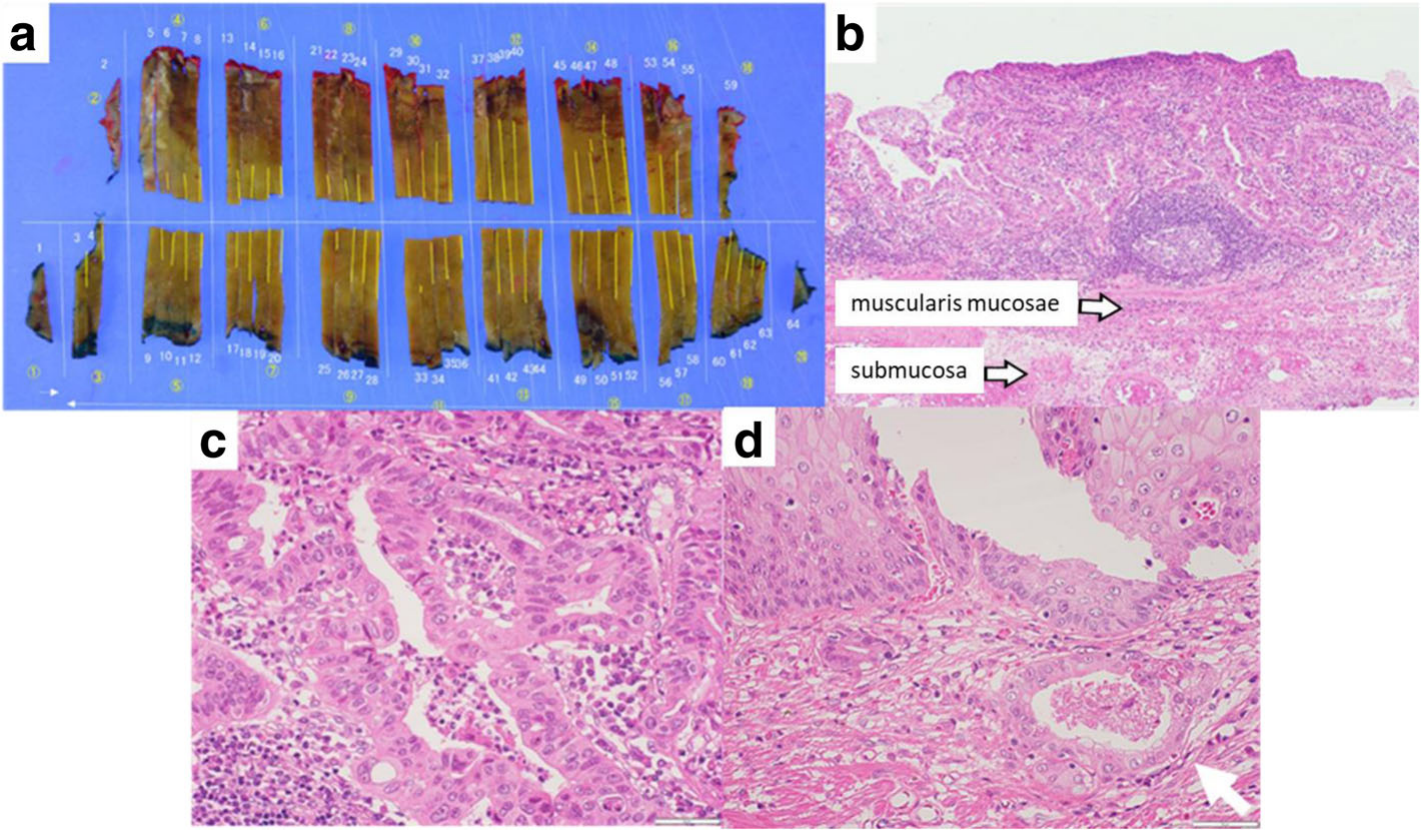
Macroscopic and histopathological findings. **a** The opened specimen measured 110 × 55 mm^2^ with the tumor occupying the whole luminal circumference. **b** The depth of tumor invasion was superficial muscularis mucosa. **c** Well-differentiated adenocarcinoma. **d** Cancer invasion observed under the adjacent squamous epithelium (arrow)

**Fig. 4:**
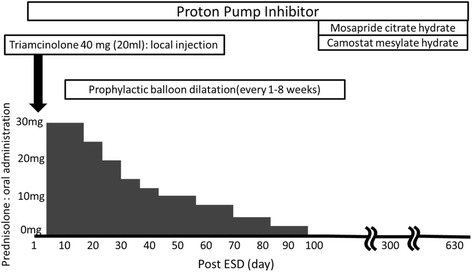
Treatment timeline

**Fig. 5:**
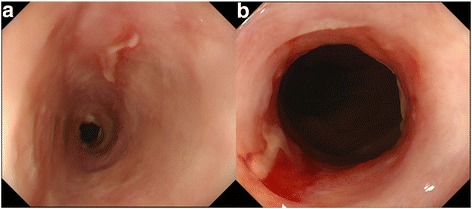
Endoscopic view of the esophagus 565 days post-ESD. **a** Linear ulcer persists. **b** The EG junction area through which an EGD (9.2-mm diameter endoscope) can be passed

A 59-year-old man complaining of heart burn for two decades was diagnosed with LSBE with an irregular surface on esophagogastroduodenoscopy (EGD) during a routine check-up. A targeted biopsy of the irregular mucosa revealed an adenocarcinoma. The patient was reported to have a 10-year history of LSBE on his annual check-ups, but no irregular mucosa in LSBE had been previously found. No remarkable medical history was elicited. Clinical examination and laboratory studies had unremarkable results. EGD showed a sliding herniation in the esophagus and LSBE was classified as C4M5 according to the Prague C & M criteria (Fig. 1a). LSBE surface mucosa was unevenly reddish and contained an erosive area (Fig. 1b-c). Narrow band imaging (NBI) with magnification revealed a scattered lesion with LSBE mucosal and vasculature irregularities (Fig. 1d-e). The biopsy revealed that most of LSBE was replaced with a well-differentiated adenocarcinoma. Endoscopic ultrasound (20 MHz) revealed thickening of the mucosal layer, although the submucosal layer was intact (Fig. 1f). No metastatic lesions were detected on enhanced CT. Finally, the patient was diagnosed with early esophageal adenocarcinoma corresponding to the LSBE area; the lesion was totally circumferential and longitudinally measured 50 mm. After receiving written informed consent, ESD was performed using an EG-580RD (Fujifilm Medical, Tokyo, Japan) with a distal attachment (D201–11804; Olympus Medical Systems, Tokyo, Japan). A submucosal injection of 0.4% sodium hyaluronate (MucoUp; Johnson and Johnson, Tokyo, Japan) was administered. Marking, incision, and dissection were performed using a hook knife (KD-620QR; Olympus Medical Systems). An electrosurgical generator (VIO300D, ERBE Elektromedizin, Tübingen, Germany) was used with specifications set at dry-cut mode 60 W, effect 3, and spray coagulation mode 60 W, effect 2. Carbon dioxide was used for insufflation. ESD procedure was performed as follows: distal marking at 5 mm caudally from the EG junction (Fig. 2a) and proximal marking 10 mm cranially from the SC junction (Fig. 2b), followed by circumferential incision at the caudal end of the lesion (Fig. 2c). A submucosal tunnel entrance was created by a partial incision at the cranial end of the lesion (Fig. 2d) and submucosal dissection was performed (Fig. 2e), followed by the creation of three submucosal tunnels in the same way (Fig. 2f). Finally, incision between the opening of each tunnel and dissection of the connective submucosal tissue between each tunnel assisted by the thread-traction method was performed. *En bloc* resection was achieved without intraoperative adverse events (Fig. 2g-h). The operation period was 12 h. Histopathological analysis of the resected tissue demonstrated that the adenocarcinoma occupied the entire lumen circumference and was well differentiated. Both the cranial and caudal margins were tumor free (Fig. 3a). The depth of the tumor invasion was the superficial muscularis mucosa without lymphatic vessel and vascular involvement (Fig. 3b-c), and minimal lateral extension under the adjacent squamous epithelium was observed (Fig. 3d). The timeline after ESD is shown in Fig. 4. Postoperative adverse events such as pneumomediastinum, mediastinitis, or bleeding did not occur. The hospitalization period was 4 weeks. An intralesional steroid injection of 40 mg triamcinolone acetonide diluted with saline to a total dose of 20 mL (40 injection points, 0.5 mL per point, 8 points per circle every 10 mm) was completed on the first day post-ESD. Oral prednisolone was administered at an initial dose of 30 mg/day on the third day post-ESD, which was gradually tapered and discontinued 14 weeks later. Prophylactic endoscopic balloon dilation (EBD) with 12–15 mm balloon diameter (CRE balloon; Boston Scientific, Boston, USA) was commenced on the 10th day post-ESD and was conducted once every 1–8 weeks for 30 weeks (20 sessions). A proton pump inhibitor (PPI), proteinase inhibitor (100 mg camostat mesylate, three times after each meal), and prokinetic agent (5 mg mosapride citrate hydrate, three times after each meal) were orally administered for almost 2 years to prevent the occurrence of GERD and promote the natural healing of the post-ESD ulcer (Fig. 4). At the 2-year follow-up, the post-ESD ulcer had been covered with regenerated squamous epithelium, although small erosions still existed at the center of the post-ESD area (Fig. 5a) and EG junction (Fig. 5b). The patient does not have dysphagia and there is no evidence of esophageal stricture or recurrence 2 years after ESD.

## Discussion and conclusions

Endoscopic mucosal resection (EMR) is a therapeutic option for esophageal intramucosal carcinoma (T1a); however, the procedure may be unsuitable for large lesions because of the difficulty associated with *en bloc* resection. *En bloc* resection is very important for an exact diagnosis and should be followed by an appropriate planned post-resection management strategy. ESD is the most common treatment method for intramucosal esophageal and gastric cancers (T1a) in Japan and facilitates *en bloc* resection even for large lesions. However, because the lesion in the present case was entirely circumferential and longitudinally measured 50 mm, treatment was presumed to be difficult even with ESD and the risk of post-ESD stricture of esophagus was extremely high. Although the alternative option of reliable treatment was open surgery, it was considered too invasive for such an early-stage Barrett’s adenocarcinoma. Therefore, we selected ESD as the treatment strategy for this lesion.

Magnifying endoscopy with NBI facilitates easy detection of early Barrett’s adenocarcinoma [3, 4]. However, the diagnosis of the extent of the tumor and attention to subsquamous tumor growth, often observed in patients with Barrett’s adenocarcinoma, is required [5]. The resected specimen revealed minimal tumor extension under the adjacent squamous epithelium. The circumferential incision of the cranial end should be undertaken at the oral side of the SC junction.

Submucosal fibrosis might exist in Barrett’s esophagus because of the preexisting inflammation of the esophageal wall. Even with ESD, dissecting a long segment of the submucosal layer with fibrosis is challenging. Most of the submucosal layer beneath the lesion showed remarkable fibrosis in the present case. Counter-traction is very important to dissect the fibrous submucosa. We initially created three submucosal tunnels. Endoscopic submucosal tunneling dissection was introduced as a safe, effective treatment for large, circumferential superficial esophageal neoplasia [6, 7]. The creation of tunnels was assisted by counter-traction produced by the surrounding fibrous submucosa. However, it was difficult to distinguish the mucosal side from the muscle side in the submucosal tunnel because both sides were whitish because of fibrosis. Therefore, the orientation of vertical direction might be lost, and this constitutes a risk factor for perforation or incision into the mucosal lesion. To maintain a vertical orientation, we frequently removed the scope from the submucosal tunnel and reconfirmed the orientation of anatomical direction. We dissected the connective submucosal tissue between each tunnel assisted by the thread-traction method [8], which was very useful for obtaining good counter-traction during the procedure.

It is well known that circumferential resection of more than three quarters of the lumen leads to esophageal stenosis. To avoid this major complication, various strategies have been implemented. However, there is no established prophylactic option for esophageal stenosis after radical resection. We employed intralesional steroid injection and oral steroid therapy [9, 10]. We frequently conducted prophylactic EBD, though it is a controversial option for preventing the esophageal stenosis [11, 12]. Frequent gastric acid and bile juice reflux was presumed in the present case because the patient suffered from esophageal hiatus herniation. Despite treatment with PPI, protease inhibitor, and prokinetic agent, squamous epithelium regeneration was slow and two erosions persisted long-term. We were concerned about the chronic inflammation with subsequent fibrosis at the ESD site. Once a firm strictural fibrosis is established, it might be difficult to relieve using therapeutic EBD. Therefore, we employed prophylactic EBD with intralesional steroid injection and oral steroid therapy. Prophylactic EBD was undertaken more often because of the slow healing of the post-ESD ulcer. To clarify the significance of prophylactic EBD with oral and intralesional steroid therapy, reports from additional cases are desirable.

ESD for LSBE mostly replaced with superficial Barrett’s adenocarcinoma (SBA) is challenging. A prophylactic approach for post-ESD esophageal stricture remains unclear. However, compared with other treatment options such as EMR and surgical operation, ESD appears to be a safe and effective option for the treatment of total circumferential long-segment Barrett’s adenocarcinoma.

## Abbreviations

EBD: Endoscopic balloon dilation
EGD: Esophagogastroduodenoscopy
EMR: Endoscopic mucosal resection
ESD: Endoscopic submucosal dissection
GERD: Gastroesophageal reflux disease
LSBE: Long-segment Barrett’s esophagus
PPI: Proton pump inhibitor
SBA: Superficial Barrett’s adenocarcinoma
